# Validating a Case Definition for Transgender Adults Using Administrative Data

**DOI:** 10.1001/jamanetworkopen.2024.51700

**Published:** 2025-01-03

**Authors:** Chantal L. Rytz, James A. King, Nathalie Saad, Paul E. Ronksley, Ranjani Somayaji, Satish R. Raj, Sandra M. Dumanski, Amelia M. Newbert, Lindsay D. Peace, Sofia B. Ahmed

**Affiliations:** 1 Cumming School of Medicine, University of Calgary, Calgary, Alberta, Canada; 2 Libin Cardiovascular Institute, University of Calgary, Calgary, Alberta, Canada; 3 Centre for Health Informatics, University of Calgary, Calgary, Alberta, Canada; 4 Provincial Research Data Services, Alberta Health Services, Calgary, Alberta, Canada; 5 Department of Medicine, University of Calgary, Calgary, Alberta, Canada; 6 Department of Community Health Sciences, University of Calgary, Calgary, Alberta, Canada; 7 O’Brien Institute for Public Health, University of Calgary, Calgary, Alberta, Canada; 8 Department of Microbiology, Immunology, and Infectious Disease, University of Calgary, Calgary, Alberta, Canada; 9 Snyder Institute for Chronic Diseases, University of Calgary, Calgary, Alberta, Canada; 10 Department of Cardiac Sciences, University of Calgary, Calgary, Alberta, Canada; 11 Skipping Stone Foundation, Calgary, Alberta, Canada; 12 Faculty of Medicine and Dentistry, University of Alberta, Edmonton, Alberta, Canada; 13 Women and Children’s Health Research Institute, University of Alberta, Edmonton, Alberta, Canada

## Abstract

**Importance:**

Administrative health data serve as promising data sources to study transgender health at a population level in the absence of self-reported gender identity.

**Objective:**

To develop and validate case definitions identifying transgender adults in administrative data compared with the reference standard of self-reported gender identity in a universal health care setting.

**Design, Setting, and Participants:**

In this cohort study conducted in Alberta, Canada, data from provincial administrative health data sources including inpatient hospitalizations, emergency department encounters, primary care visits, prescription drug dispensations, and the provincial health insurance registry were linked and used to develop 15 case definitions (9 for transgender women and 6 for transgender men). Participants aged 18 years or older with a provincial health care number between April 1, 1994, and March 31, 2021, were included and stratified by sex marker (eg, female or male) at study entry. Data analysis was from December 2023 to March 2024.

**Main Outcomes and Measures:**

For each case definition, the sensitivity, specificity, positive predictive value (PPV), and negative predictive value (NPV) were calculated against the reference standard of self-reported gender identity.

**Results:**

In this cohort study of 5 375 735 individuals, the reference standard consisted of 141 self-identified transgender women, 174 self-identified transgender men, 111 self-identified cisgender women, and 65 self-identified cisgender men. The final cohort representing transgender women participants who met at least 1 case definition and/or were part of the standard reference totaled 63 977. Combining a case definition employing male sex registry identification and 2 or more dispensations of estrogen or a case definition employing male sex registry identification and at least 1 gender-related diagnostic code demonstrated a sensitivity of 86.6% (95% CI, 79.9%-91.7%), specificity of 62.5% (95% CI, 51.5%-72.6%), PPV of 78.8% (95% CI, 71.6%-85.0%), and NPV of 74.3% (95% CI, 62.8%-83.8%). The final cohort representing transgender men participants who met at least 1 case definition and/or were part of the standard reference totaled 26 852. Combining a case definition employing female sex registry identification and 2 or more dispensations of testosterone or a case definition employing female sex registry identification and at least 1 gender-related diagnostic code demonstrated a sensitivity of 78.2% (95% CI, 71.3%-84.1%), specificity of 89.2% (95% CI, 82.2%-94.1%), PPV of 91.3% (95% CI, 85.5%-95.3%), and NPV of 73.8% (95% CI, 65.8%-80.7%).

**Conclusion and Relevance:**

These findings suggest that case definitions using transgender-related diagnostic codes and gender-affirming hormone prescriptions can be used to study the epidemiology, disease burden, and health care utilization of transgender populations.

## Key Points

**Question:**

How effective are case definitions in identifying transgender adults within administrative health data compared with self-reported gender identity in a universal health care setting?

**Findings:**

In this cohort study of 5 375 735 individuals, 9 transgender women and 6 transgender men case definitions were created using provincial administrative health data sources (1994-2021) including inpatient hospitalizations, emergency department encounters, primary care visits, prescription medications, and provincial insurance registry information. Case definitions using transgender-related diagnostic codes and gender-affirming hormone prescriptions demonstrated the highest sensitivity compared with other algorithms.

**Meaning:**

These findings suggest that in the absence of self-reported gender identity, these validated case definitions can be useful to evaluate health care needs of transgender populations.

## Teaser

This cohort study develops case definitions for transgender men and women and compares their validity with the reference standard of self-reported gender identity among individuals in Alberta, Canada.

## Introduction

Transgender individuals face important health challenges^1^ but have been historically underserved by health care and research.^2,3,4^ Establishing the prevalence, disease burden, and unique health needs of transgender communities remains difficult^3^ because there are currently no mechanisms in place to determine health care gaps at a population level.

Administrative health data provide an opportunity to explore epidemiological questions across populations, and have been highlighted as a way to improve health care delivery,^5^ evidence-based decision-making,^6^ and clinical outcomes specifically in the transgender population.^7^ However, use of administrative data is currently limited in transgender populations due to conflation of sex and gender terms,^8^ sex and gender binary–focused health data collection,^9^ and variability in the definition of *transgender*,^10^ as well as social stigma impeding reporting and health documentation. This has been exacerbated by inadequate information exchange between patients and clinicians,^11^ lack of patient consent to disclose transgender identity, and information requirements that may conflict with patient preferences or legal obligations,^12^ thereby constraining gender identity ascertainment with resulting erasure of transgender individuals in health research and care.^13^ Numerous case definitions to identify transgender individuals in US administrative data have been created to address this need,^14,15^ although not all have been validated against self-identified gender identity. Case definitions to identify transgender adults in administrative data using transgender-related diagnosis and procedure codes and gender-affirming hormone therapy have been demonstrated to perform well in a US-based comprehensive federally qualified community health center offering integrated primary medical and behavioral health services with an emphasis on lesbian, gay, bisexual, and transgender health.^16^ However, how well these case definitions perform in a general population and in a universal health care system^17^ is unknown. Previous studies using case definitions for identification of transgender individuals in provincial jurisdictions in Canada have focused on populations with specific chronic conditions^18^ or did not use validation methods to determine their accuracy.^19^ The importance of accurate gender identity data collection^20^ to address and improve health outcomes in the transgender population^21,22^ led to the creation and validation of case definitions to identify transgender women and men in a population-based cohort.

## Methods

### Persons With Living Experience-Oriented Research Approach

This project focused on a knowledge gap and research priority identified by individuals and community organizations with living experience. Individuals from the transgender community were members of our research team and participated in the conceptualization, case definition design, manuscript framework, and writing of this work.

### Study Design

This retrospective cohort study used deidentified, population-based administrative data from the province of Alberta, Canada (population of approximately 4.9 million). The University of Calgary Conjoint Health Research Ethics Board approved this study, and a waiver of informed consent to extract health information under the conditions of the Health Information Act (Alberta, Canada) was granted. Investigators had no way of contacting or identifying individuals included in this cohort. This analysis was reported according to the Reporting of Studies Conducted Using Observational Routinely Collected Data (RECORD)^23^ and Strengthening the Reporting of Observational Studies in Epidemiology (STROBE) reporting guidelines.

### Data Sources

Data sources included the Provincial Health Insurance Registry, Physician Claims Database, National Ambulatory Care Classification Database, Discharge Abstract Database, and data on dispensed prescription medication from the Pharmaceutical Information Network, which collects information from all outpatient pharmacies in the province of Alberta. These databases were originally created for health care management and monitoring (eg, insurance claims and remunerating physicians) under the universal health care system in Canada, yet are suitable for research purposes because they contain detailed information at the population level and have mechanisms to ensure high data quality.^24,25,26^ These data in Alberta are maintained by Alberta Health Services and cover more than 99.0% of the general population of the province. Deterministic linkage using the personal health number, sex marker, and date of birth were used to link records from multiple datasets internally by an Alberta Health Services analyst (J.A.K.). Case definitions utilized diagnostic coding from inpatient encounters (up to 25 diagnosis codes, based on the *International Statistical Classification of Diseases and Related Health Problems, 10th Revision, Canada [ICD-10-CA]*), emergency and ambulatory care encounters (up to 10 diagnosis codes, based on the *International Statistical Classification of Diseases and Related Health Problems, Tenth Revision [ICD-10)]*), and physician billing claim data (up to 3 diagnosis codes, based on the *International Classification of Diseases, Ninth Revision, Clinical Modification [ICD-9-CM]*). Procedure codes for certain case definitions were linked to inpatient, emergency, and ambulatory databases based on the Canadian Classification of Health Interventions (up to 20 codes in inpatient settings and up to 10 codes in emergency or ambulatory settings). These records were extracted from April 1, 2008, to March 31, 2021.

### Identification of Study Cohort

Participants aged 18 years or older with an Alberta provincial health care number between April 1, 1994, and March 31, 2021, were included in the cohort. Participants were stratified by the sex marker (eg, female or male) listed at study entry. Nine operational case definitions for transgender women and 6 operational case definitions for transgender men were developed by previous publications^16,18,19,27^ and supplemented by input from a team of persons with living experience, gender-affirming care clinicians, and researchers with gender medicine and administrative database expertise (eTable 1 in Supplement 1). Case definition algorithms used a combination of *ICD-9-CM* and *ICD-10-CA* codes for diagnoses related to gender dysphoria, procedural codes for gender-affirming surgery, change in registered sex marker, and drug identification numbers for commonly filled prescriptions for medical transition (eg, estradiol, progestin, and antiandrogens for participants with male sex marker or testosterone for participants with female sex marker). If individuals were identified by multiple case definitions, the case definition with the earliest date was used as the index date. Case definitions that identified fewer than 5 participants as transgender were excluded to preserve anonymity. The reference standard was created by physician-led chart review to record the self-reported gender identity of randomly selected patients who attended 1 endocrinology clinic in Calgary, Canada from January 1, 2013, to March 31, 2021.

### Statistical Analyses

Validity indices including sensitivity, specificity, positive predictive value (PPV), and negative predictive value (NPV) were calculated against self-identified gender identity for each case definition. Receiver operator curve areas were calculated with 95% CIs. Given the low documented prevalence of transgender persons in Canada,^28^ sensitivity was the a priori primary criterion for determining the best-performing case definition. Population-level analyses were conducted to determine prevalence and incidence per 100 000 person-years and were graphically generated to explore temporal changes across a 10-year period. All analyses were performed using Stata version 16 (StataCorp) and graphs were generated using R version 4.4.3 (R Project for Statistical Computing). Data analysis was conducted from December 2023 to March 2024.

## Results

**Figure 1.**
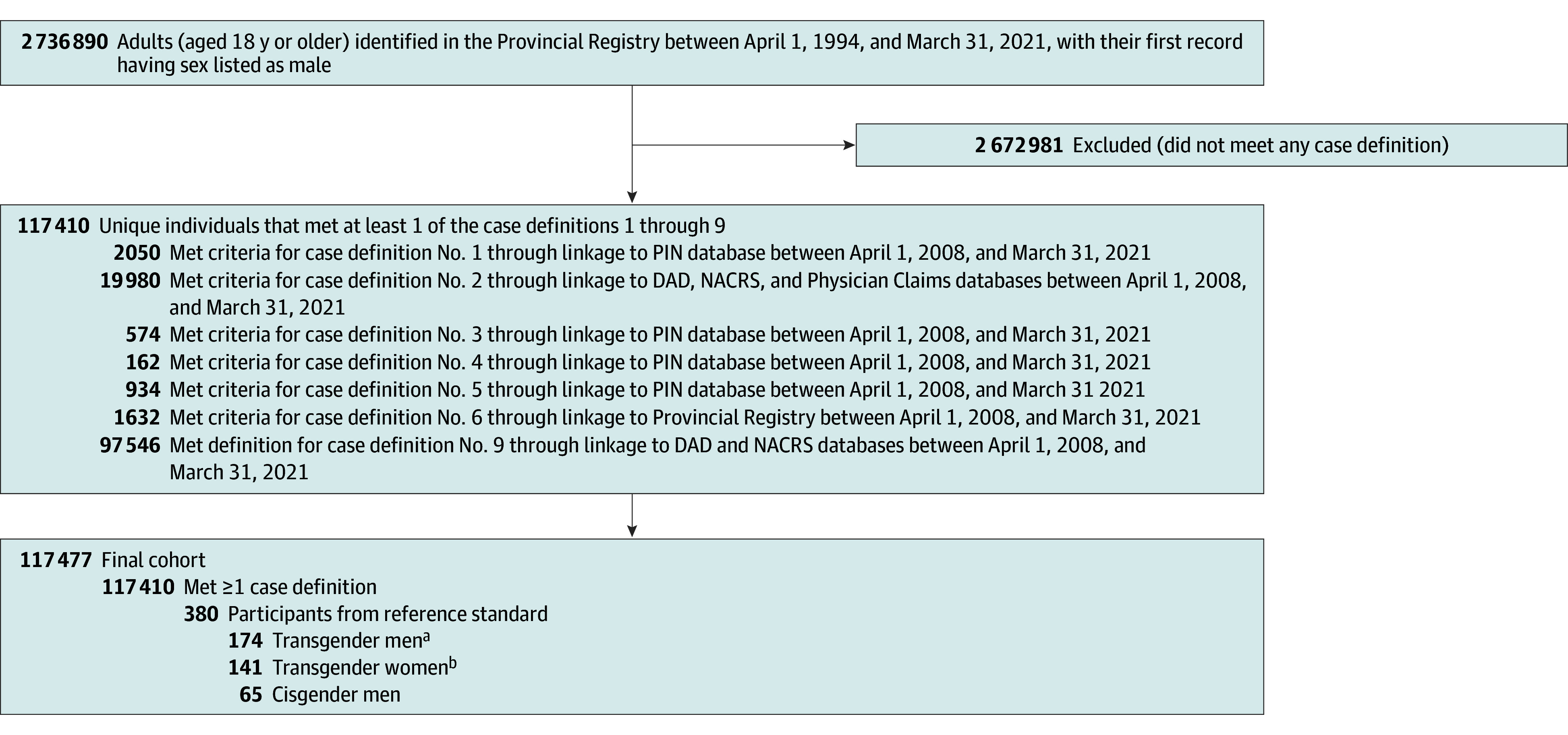
Flowchart for the Identification of Transgender Women DAD indicates Discharge Abstract Database; NACRS, National Ambulatory Care Reporting System; PIN, Pharmaceutical Information Network. ^a^Transgender men from the reference standard who were identified by any case definition were included. ^b^Self-identified transgender women from the reference standard who were not identified by a case definition were added to final cohort.

**Figure 2.**
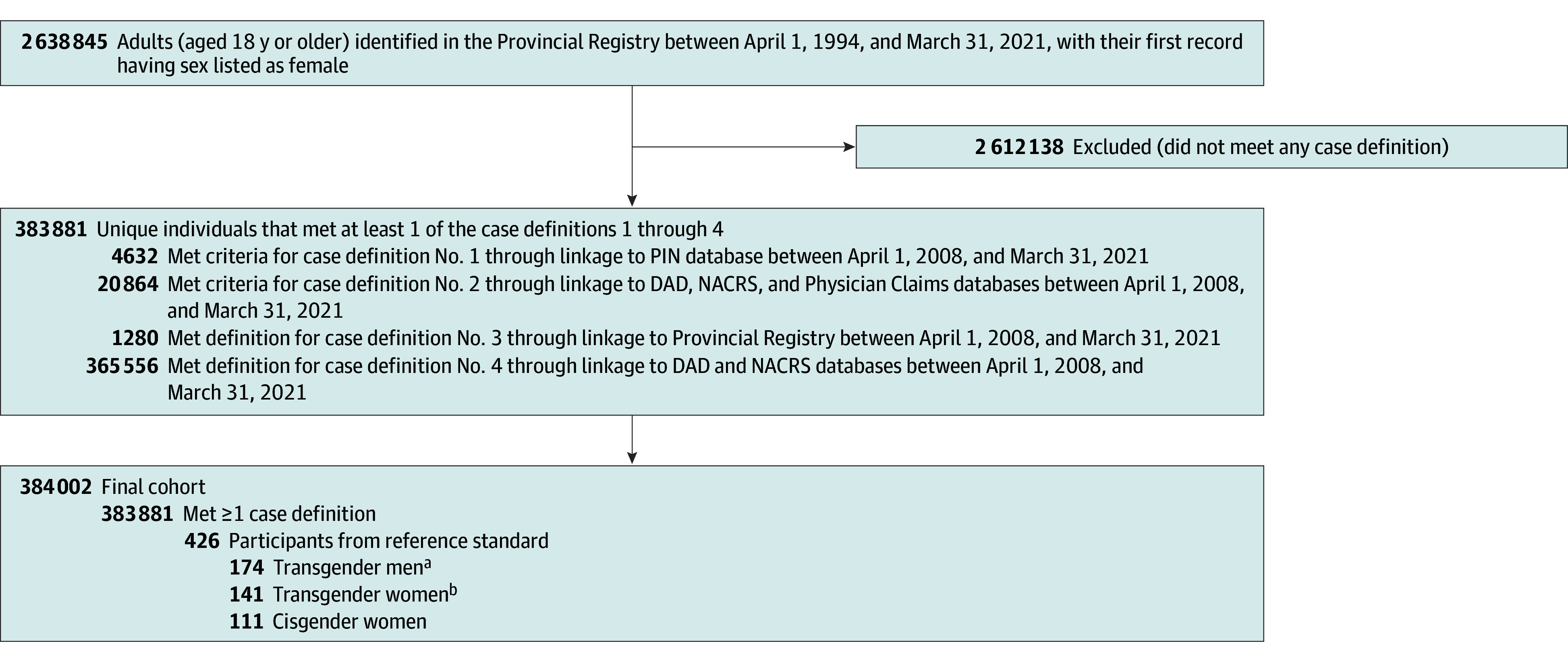
Flowchart for the Identification of Transgender Men DAD indicates Discharge Abstract Database; NACRS, National Ambulatory Care Reporting System; PIN, Pharmaceutical Information Network. ^a^Self-identified transgender men from the reference standard who were not identified by a case definition were added to final cohort. ^b^Transgender women from the reference standard who were identified by any case definition were included.

A total of 5 375 735 individuals with a provincial health care number between April 1, 1994, and March 31, 2021, were included. Each of the 15 case definitions, including the data sources and identifiers used, are described in eTable 1 in Supplement 1. A flow-chart of the data linkage process and cohort generation process, including the number of individuals identified at each step by each case definition for transgender women and men are shown in Figure 1 and Figure 2, respectively. The sensitivities and PPV of each case definition tested against the reference standard of self-identified transgender individuals are described in eTable 2 in Supplement 1, and case definition performance tested against the reference standard of self-identified transgender women (141 transgender women) and self-identified transgender men (174 transgender men; ie, true positives) and self-identified cisgender women (111 cisgender women) and self-identified cisgender men (65 cisgender men; ie, true negatives) are presented in the Table.

**Table.** Case Definition Performance in Identification of Transgender Women and Men Compared With Self-Identified Transgender and Cisgender Reference Standard

Case definition	Sensitivity, % (95% CI)	Specificity, % (95% CI)	Positive predictive value, % (95% CI)	Negative predictive value, % (95% CI)	Receiver operator curve area (95% CI)
Transgender women					
1^a^	71.8 (63.7-79.1)	98.9 (93.9-100.0)	99.0 (94.7-100.0)	68.5 (59.7-76.5)	0.85 (0.81-0.89)
2^b^	85.2 (78.3-90.6)	62.5 (51.5-72.6)	78.6 (71.2-84.8)	72.4 (60.9-82.0)	0.74 (0.68-0.80)
1 and 2^a^^,^^b^	70.4 (62.2-77.8)	98.9 (93.9-100.0)	99.0 (94.6-100.0)	67.4 (58.6-75.4)	0.85 (0.81-0.89)
1 or 2^a^^,^^b^	86.6 (79.9-91.7)	62.5 (51.5-72.6)	78.8 (71.6-85.0)	74.3 (62.8-83.8)	0.75 (0.69-0.80)
Transgender men					
1^c^	62.6 (55.0-69.8)	100.0 (97.0-100.0)	100.0 (96.7-100.0)	64.9 (57.5-71.7)	0.81 (0.78-0.85)
2^d^	77.0 (70.0-83.0)	89.2 (82.2-94.1)	91.2 (85.4-95.2)	72.8 (64.8-79.8)	0.83 (0.79-0.87)
1 and 2^c^^,^^d^	61.5 (53.8-68.8)	100.0 (97.0-100.0)	100.0 (96.6-100.0)	64.2 (56.8-71.0)	0.81 (0.77-0.84)
1 or 2^c^^,^^d^	78.2 (71.3-84.1)	89.2 (82.2-94.1)	91.3 (85.5-95.3)	73.8 (65.8-80.7)	0.84 (0.80-0.88)

^a^ Male and 2 or more estrogen prescriptions.

^b^ Male and 1 or more gender-related *International Classification of Diseases, Ninth Revision, Clinical Modification* (*ICD-9-CM*); *International Statistical Classification of Diseases and Related Health Problems, 10th Revision, Canada* (*ICD-10-CA*); or *International Statistical Classification of Diseases, Tenth Revision *(*ICD-10*) codes.

^c^ Female and 2 ore more testosterone prescriptions.

^d^ Female and 1 or more gender-related *International Classification of Diseases, Ninth Revision, Clinical Modification* (*ICD-9-CM*); *International Statistical Classification of Diseases and Related Health Problems, 10th Revision, Canada* (*ICD-10-CA*); or *International Statistical Classification of Diseases, Tenth Revision *(*ICD-10*) codes.

### Transgender Women

A total of 2 736 890 participants with a male sex marker were identified. Nine transgender women–specific case definitions were generated (eTable 1 in Supplement 1), and the final cohort representing participants who met at least 1 case definition and/or were part of the standard reference totaled 117 477 (Figure 1). Case definition performance against transgender women–only referents resulted in sensitivities ranging from 0.7% (95% CI, 0.0%-3.9%) to 85.2 (78.3%-90.6%) and PPV ranging from 0.0% (95% CI, 0.0%-0.2%) to 5.0 (eTable 2 in Supplement 1). Case definitions 1 (male sex marker and ≥2 dispensations of exogenous estrogen) and 2 (male sex marker and at least 1 gender-related *ICD-9-CM* or *ICD-10* diagnostic code) each demonstrated high sensitivity and low PPV (eTable 2 in Supplement 1). The overall performance within the entire reference standard of both transgender and cisgender individuals is outlined in the Table. Case definition 1 and case definition 2 performed comparably, and there was considerable overlap of participants who met criteria for both aforementioned case definitions (44 784 participants [70.0%]). The combination of case definition 1 or case definition 2 demonstrated a sensitivity of 86.6% (95% CI, 79.9%-91.7%), specificity of 62.5% (95% CI, 51.5%-72.6%), PPV of 78.8% (95% CI, 71.6%-85.0%), and NPV of 74.3% (95% CI, 62.8%-83.8%). A sensitivity analysis of the false positives identified by combining case definitions 1 and 2 revealed that the majority (33 of 47 false positives [70.2%]) were transgender men, suggesting misclassification bias.

### Transgender Men

A total of 2 638 845 participants with a female sex marker were identified. Six transgender men–specific case definitions were generated (eTable 1 in Supplement 1), and the final cohort representing participants who met at least 1 case definition and/or were part of the standard reference totaled 384 002 (Figure 2). Case definition performance against transgender men–only referents resulted in sensitivities ranging from 0.6% (95% CI, 0.0%-3.2%) to 77.0% (95% CI, 70.0%-83.0%) and PPV ranging from 0.0% (95% CI, 0.0%-0.0%) to 2.7% (95% CI, 1.7%-3.9%) (eTable 2 in Supplement 1). Case definitions 1 (female sex marker and ≥2 dispensations of exogenous testosterone) and 2 (female sex marker and at least 1 gender-related *ICD-9-CM* or *ICD-10* diagnostic code) each demonstrated high sensitivity and low PPV (eTable 2 in Supplement 1). The overall performance within the entire reference standard is outlined in the Table. Case definition 1 and case definition 2 performed comparably, and there was considerable overlap of participants who met criteria for both case definition 1 and 2 (16 380 participants [61.0%]). A combination of case definitions 1 or 2 demonstrated a sensitivity of 78.2% (95% CI, 71.3%-84.1%), specificity of 89.2% (95% CI, 82.2%-94.1%), PPV of 91.3% (95% CI, 85.5%-95.3%), and NPV of 73.8% (95% CI, 65.8%-80.7%). A sensitivity analysis of the false positives identified by combining case definitions 1 and 2 revealed that the majority (13 of 19 false positives [68.4%]) were transgender women, suggesting misclassification bias.

### Incidence and Prevalence of Transgender Women and Men in Alberta

The incidence of transgender women and transgender men were assessed independently using both case definition 1 and case definition 2, as well as a combination of either case definition (eFigure 1 and eFigure 2 in Supplement 1). The incidence per 100 000 person-years is described for both transgender women and transgender men in eTable 3 in Supplement 1. Depending on the case definition used and the year of observation, the incidence per 100 000 person-year varied from 1.5 (95% CI, 1.1-2.0) to 60.2 (95% CI, 57.6-62.9) for transgender women (eFigure 1 in Supplement 1), and 1.1 (95% CI, 0.8-1.6) to 64.9 (95% CI, 62.0-67.9) for transgender men (eFigure 2 in Supplement 1). The combined (ie, hormone therapy and/or diagnostic coding) case definitions in our study suggested a prevalence of 0.48% transgender women (20 336 of 4 262 635 individuals), 0.56% transgender men (23 881 of 4 262 635 individuals), and an overall estimated prevalence of 1.04% transgender individuals (44 217 of 4 262 635 individuals) in Alberta in 2021.

## Discussion

In this cohort study, we assessed the validity of administrative data for defining transgender adults compared with the reference standard of self-identified gender identity in a general population in a publicly funded universal health care system. The combined (ie, hormone therapy and/or *ICD-9-CM* or *ICD-10* coding) case definitions in our study suggested a prevalence of 0.48% transgender women, 0.56% transgender men, and an overall estimated prevalence of 1.04% transgender individuals in Alberta. Our results suggest that employing a combination of sex marker and either prescriptions for gender-affirming hormone therapy or transgender-specific diagnostic codes represents a feasible identification strategy for both transgender women and transgender men and may be useful for defining cohorts or adjustment for gender identity in a research setting.

The 2022 National Academies of Sciences, Engineering, and Medicine report of Measuring Sex, Gender Identity, and Sexual Orientation^8^ highlighted the specific and urgent need for improving monitoring health and health care disparities to ultimately achieve health equity in this population. In the 2021 Canadian Census of Population,^28^ questions regarding both gender identity and sex assigned at birth were included for the first time, acknowledging Canada as the first country to collect and publish data on gender diversity from a national census. Previous studies have also developed case definitions to identify transgender individuals in health care data.^15,16,18,29,30,31,32,33^ The combination of sexual and gender identity fields and *ICD-10* codes correctly identified more than 99% of the gender-expansive population in a study conducted in a single academic center.^30^ Similarly, a 2023 study^16^ developed a case definition comprised of *International Classification of Diseases, Ninth Revision (ICD-9)* or *ICD-10 *diagnosis codes, surgical billing codes or documentation of gender-affirming surgeries, and prescription data correctly categorized 87.3% of transgender adults and 98.7% of cisgender adults using self-reported gender identity as a benchmark at a large federally qualified community health center. Despite underscoring the usability of administrative data to advance transgender health, these studies^16,30^ utilized records from single medical centers serving as regional centers for specialized gender-affirming care, which may limit the generalizability of results to an unselected general population as addressed in our study design.

Previous work has highlighted the benefits of using unstructured data (eg, keywords or free-text notes) in combination with diagnostic codes. In a 2016 study,^15^ a case definition was developed and validated to identify transgender women and men using a 3-step algorithm derived from electronic medical records of insurance-based health care clinics, where 99% of individuals were identified using unstructured data (eg, keywords or free-text notes) and/or *ICD-9*, whereas only 9% were identified with *ICD-9* codes alone.^15^ Similarly, in a 2021 study,^29^ transgender case definitions developed using electronic health records and unstructured clinical notes resulted in high sensitivity (42.6%) using 2 or *ICD-9-CM *or *International Statistical Classification of Diseases, Tenth Revision, Clinical Modification (ICD-10-CM)* diagnosis codes, although the overall performance improved with the addition of unstructured data (specificity, 99.5%; PPV, 96.7%; NPV, 82.7%). However, while the incorporation of unstructured data may improve the performance of case definitions^34,35^ and has been previously employed in case definitions,^15,29,32^ there remains a need for further research and guideline development on best practices to use and integrate unstructured data in health research,^36^ highlighting the importance of developing case definitions using administrative data.

Other case definitions to identify transgender individuals have been published in publicly funded health care settings. In Rich et al,^18^ a computable phenotype was developed to identify the proportion of transgender people within the HIV-positive population in the province of British Columbia, Canada compared with clinician-reported transgender status. In partial agreement with our findings, the best performing case definitions employed 1 or more transgender-related *ICD-9* or *ICD-10* diagnoses and 1 or more instances of androgen blocker or hormone prescription use ever, although they demonstrated lower sensitivity (27.5%; 95% CI, 17.8%-39.8%), higher specificity (99.8%; 95% CI, 99.6%-99.8%), and higher PPV (43.2%; 95% CI, 28.7%-58.9%)^18^ as compared with our top-performing case definition; these differences may reflect the differences in populations in the Rich et al^18^ study vs our study (eg, HIV-specific vs unselected general population). Finally, case definitions have been developed in the province of Saskatchewan, Canada to identify transgender individuals using *ICD-9* or *ICD-10-CA *diagnosis, billing claims, or prescription drug dispensations, although these case definitions were not validated, and therefore their reliability and accuracy are unknown.^19^

### Limitations and Strengths

This study has limitations. First, the best-performing case definitions require individuals to access health care for gender dysphoria–related diagnoses or gender-affirming hormone therapy, which would not capture transgender individuals who do not fit these criteria. In a recent national survey,^37^ only one-half of transgender participants in Alberta had a primary care clinician with whom they felt mostly or very comfortable discussing transgender health issues. However, a large percentage of transgender individuals in Canada undergo medical gender affirmation,^38^ with a high 4-year continuation rate among transgender women and men.^39^ Moreover, the top performing algorithms in this study are the same as the optimal case definitions identified in similar studies,^16,18^ and the performance of the case definitions in our study is similar to that of other validated algorithms.^40^ Although this may have underestimated the prevalence of transgender individuals within the study population,^41^ the case definitions in our study suggested a prevalence of 0.48% for transgender women and 0.56% for transgender men. Furthermore, our overall estimated prevalence (1.04%) of the transgender population in Alberta was higher than that reported in other Canadian provinces (0.4% to 0.5%),^18,19^ and in the 2021 Canadian Census (0.33% in Canada; 0.30% in Alberta),^28^ but in agreement with census results on individuals aged 15 to 34 years,^28^ which is similar to the age of transgender women and transgender men in our reference standard. Of note, the population in Alberta is the youngest in Canada,^42^ and the prevalence of transgender individuals is higher among younger compared with older populations.^43,44,45^ These prevalence discrepancies may reflect proxy responses in census data as well as a reluctance to self-report transgender status in governmental surveys due to concerns and stigma around how this information will be used.^46^ Second, the PPV values in our study were low. However, because PPV inherently varies with pretest probability, it is not unexpected given the low documented prevalence of transgender persons in Canada.^28^ Next, we were unable to ascertain if the sex marker in the database reflected the individual’s sex listed on their birth certificate, their legal sex, or their experiential sex; given the lack of standardization of how this variable was collected, the reliability is uncertain. We made efforts to assess a variety of case definitions acknowledging the spectrum of gender affirmation approaches, including scenarios not reliant on medical-focused transition processes (eg, sex marker changes). However, because electronic health records predominantly report sex and/or gender in a binary fashion, it is possible that intersex individuals; individuals with disorders of sexual development; or cisgender, gender-diverse, and nonbinary individuals using hormone therapy were inappropriately identified as transgender, and transgender individuals with nonlinear gender affirmation processes, or gender-diverse and nonbinary individuals may have been inappropriately identified as cisgender, thus introducing confounding in analyses. Implementation of data collection practices accurately capturing self-reported gender identity data, as recommended by the Canadian Institute for Health Information,^47^ is urgently needed to address these concerns. Additionally, as in all case definitions based on administrative data, the results may be less generalizable to other jurisdictions where there may be differences in coding for claims and hospitalizations.

This study also has strengths. The use of computerized prescription drug data eliminates any potential impact of recall bias by study participants. The size of the population-based cohort with almost 30 years of follow-up increases the generalizability of our findings. As far as we are aware, this is the first study to evaluate case definition performance against the reference standard of self-reported gender identity in an unselected population in a universal health care setting.

## Conclusions

In conclusion, our study represents a robust methodological approach to develop and validate case definitions identifying transgender adults in administrative data compared with the reference standard of self-reported gender identity in a universal health care setting of the general population. Transgender individuals are frequently excluded within health research due to limited sample size, inadequate research designs, and other methodological and institutional erasure practices. Our findings represent a promising opportunity to create much-needed evidence to optimize the health care of the transgender population.

## Notes

Supplement 1. eTable 1. Data Sources and Identifiers for Case Definitions to Identify Transgender Women and Men in Alberta eTable 2. Case Definition Performance in Identification of Transgender Women and Men in Self-Identified Transgender Cohort eFigure 1. Incidence per 100,000 Person-Years for Transgender Women, by Case Definition eFigure 2. Incidence per 100,000 Person-Years for Transgender Men, by Case Definition eTable 3. Incidence of Transgender Women and Men per 100,000 Person-Years Over a 10-Year Period, by Case Definition

Supplement 2.
Data Sharing Statement

## References

[1] Streed CG Jr, McCarthy EP, Haas JS. Association between gender minority status and self-reported physical and mental health in the United States. JAMA Intern Med. 2017;177(8):1210-1212. doi:10.1001/jamainternmed.2017.1460 28558100 PMC5818796

[2] Veale JF, Deutsch MB, Devor AH, . Setting a research agenda in trans health: an expert assessment of priorities and issues by trans and nonbinary researchers. Int J Transgend Health. 2022;23(4):392-408. doi:10.1080/26895269.2022.2044425 36324879 PMC9621229

[3] Reisner SL, Poteat T, Keatley J, . Global health burden and needs of transgender populations: a review. Lancet. 2016;388(10042):412-436. doi:10.1016/S0140-6736(16)00684-X 27323919 PMC7035595

[4] Reisner SL, Conron KJ, Tardiff LA, Jarvi S, Gordon AR, Austin SB. Monitoring the health of transgender and other gender minority populations: validity of natal sex and gender identity survey items in a U.S. national cohort of young adults. BMC Public Health. 2014;14(1):1224. doi:10.1186/1471-2458-14-1224 25427573 PMC4289327

[5] Campanella P, Lovato E, Marone C, . The impact of electronic health records on healthcare quality: a systematic review and meta-analysis. Eur J Public Health. 2016;26(1):60-64. doi:10.1093/eurpub/ckv122 26136462

[6] Kruse CS, Mileski M, Vijaykumar AG, Viswanathan SV, Suskandla U, Chidambaram Y. Impact of electronic health records on long-term care facilities: systematic review. JMIR Med Inform. 2017;5(3):e35. doi:10.2196/medinform.7958 28963091 PMC5640822

[7] Reisner SL, Deutsch MB, Bhasin S, . Advancing methods for US transgender health research. Curr Opin Endocrinol Diabetes Obes. 2016;23(2):198-207. doi:10.1097/MED.0000000000000229 26845331 PMC4916925

[8] National Academies of Science Engineering and Medicine. Measuring Sex, Gender Identity, and Sexual Orientation. 2022. Accessed November 7, 2024. https://nap.nationalacademies.org/catalog/26424/measuring-sex-gender-identity-and-sexual-orientation 35286054

[9] Deutsch MB, Green J, Keatley J, Mayer G, Hastings J, Hall AM; World Professional Association for Transgender Health EMR Working Group. Electronic medical records and the transgender patient: recommendations from the World Professional Association for Transgender Health EMR Working Group. J Am Med Inform Assoc. 2013;20(4):700-703. doi:10.1136/amiajnl-2012-001472 23631835 PMC3721165

[10] Collin L, Reisner SL, Tangpricha V, Goodman M. Prevalence of transgender depends on the “case” definition: a systematic review. J Sex Med. 2016;13(4):613-626. doi:10.1016/j.jsxm.2016.02.001 27045261 PMC4823815

[11] Rytz CL, Pattar BSB, Mizen SJ, . Transgender and nonbinary individuals’ perceptions regarding gender-affirming hormone therapy and cardiovascular health: a qualitative study. Circ Cardiovasc Qual Outcomes. 2024;17(9):e011024. doi:10.1161/CIRCOUTCOMES.124.011024 39022828

[12] Rytz CL, Ahmed SB. Inclusive laboratory reference intervals and clinical studies to reduce health disparities. Clin Lab Med. 2024;44(4):563-573. doi:10.1016/j.cll.2024.07.008 39490116

[13] Bauer GR, Hammond R, Travers R, Kaay M, Hohenadel KM, Boyce M. “I don’t think this is theoretical; this is our lives”: how erasure impacts health care for transgender people. J Assoc Nurses AIDS Care. 2009;20(5):348-361. doi:10.1016/j.jana.2009.07.004 19732694

[14] Jasuja GK, de Groot A, Quinn EK, . Beyond gender identity disorder diagnoses codes: an examination of additional methods to identify transgender individuals in administrative databases. Med Care. 2020;58(10):903-911. doi:10.1097/MLR.0000000000001362 32925416 PMC8010422

[15] Roblin D, Barzilay J, Tolsma D, . A novel method for estimating transgender status using electronic medical records. Ann Epidemiol. 2016;26(3):198-203. doi:10.1016/j.annepidem.2016.01.004 26907539 PMC4772142

[16] Streed CG, King D, Grasso C, . Validation of an administrative algorithm for transgender and gender diverse persons against self-report data in electronic health records. J Am Med Inform Assoc. 2023;30(6):1047-1055. doi:10.1093/jamia/ocad039 36921287 PMC10198536

[17] Ridic G, Gleason S, Ridic O. Comparisons of health care systems in the United States, Germany and Canada. Mater Sociomed. 2012;24(2):112-120. doi:10.5455/msm.2012.24.112-120 23678317 PMC3633404

[18] Rich AJ, Poteat T, Koehoorn M, . Development of a computable phenotype to identify a transgender sample for health research purposes: a feasibility study in a large linked provincial healthcare administrative cohort in British Columbia, Canada. BMJ Open. 2021;11(3):e040928. doi:10.1136/bmjopen-2020-040928 33766836 PMC7996659

[19] Rose G, Goalen S, Clark M, Madill S. Identifying a cohort of people who are transgender and gender-diverse within Saskatchewan’s administrative health databases. Health Serv Insights. 2024;17:11786329231222122. Published online January 10, 2024. doi:10.1177/11786329231222122 38223213 PMC10785721

[20] Cahill S, Makadon H. Sexual orientation and gender identity data collection in clinical settings and in electronic health records: a key to ending LGBT health disparities. LGBT Health. 2014;1(1):34-41. doi:10.1089/lgbt.2013.0001 26789508

[21] Deb B, Porter K, van Cleeff A, Reardon LC, Cook S. emphasizing sexual orientation and gender identity data capture for improved cardiovascular care of the LGBTQ+ population. JAMA Cardiol. 2024;9(3):295-302. doi:10.1001/jamacardio.2023.5267 38265768

[22] Tran NK, Rosendale N, Lunn MR. Advancing data collection of sexual orientation and gender identity in cardiology. JAMA Cardiol. 2024;9(3):206-208. doi:10.1001/jamacardio.2023.5264 38265839

[23] Benchimol EI, Smeeth L, Guttmann A, ; RECORD Working Committee. The reporting of studies conducted using observational routinely-collected health data (RECORD) statement. PLoS Med. 2015;12(10):e1001885. doi:10.1371/journal.pmed.1001885 26440803 PMC4595218

[24] Quan H, Khan N, Hemmelgarn BR, ; Hypertension Outcome and Surveillance Team of the Canadian Hypertension Education Programs. Validation of a case definition to define hypertension using administrative data. Hypertension. 2009;54(6):1423-1428. doi:10.1161/HYPERTENSIONAHA.109.139279 19858407

[25] Ronksley PE, Tonelli M, Quan H, ; Alberta Kidney Disease Network. Validating a case definition for chronic kidney disease using administrative data. Nephrol Dial Transplant. 2012;27(5):1826-1831. doi:10.1093/ndt/gfr598 22015442

[26] Cairncross ZF, Nelson G, Shack L, Metcalfe A. Validation in Alberta of an administrative data algorithm to identify cancer recurrence. Curr Oncol. 2020;27(3):e343-e346. doi:10.3747/co.27.5861 32669943 PMC7339860

[27] Getahun D, Nash R, Flanders WD, . Cross-sex hormones and acute cardiovascular events in transgender persons: a cohort study. Ann Intern Med. 2018;169(4):205-213. doi:10.7326/M17-2785 29987313 PMC6636681

[28] Statistics Canada. Canada is the first country to provide census data on transgender and non-binary people. Published April 27, 2022. Updated May 31, 2023. Accessed November 9, 2023. https://www150.statcan.gc.ca/n1/daily-quotidien/220427/dq220427b-eng.htm

[29] Guo Y, He X, Lyu T, . Developing and validating a computable phenotype for the identification of transgender and gender nonconforming individuals and subgroups. AMIA Annu Symp Proc. 2021;2020:514-523. 33936425 PMC8075543

[30] Hines NG, Greene DN, Imborek KL, Krasowski MD. Patterns of gender identity data within electronic health record databases can be used as a tool for identifying and estimating the prevalence of gender-expansive people. JAMIA Open. 2023;6(2):ooad042. doi:10.1093/jamiaopen/ooad042 37359949 PMC10290553

[31] Wolfe HL, Reisman JI, Yoon SS, . Validating data-driven methods for identifying transgender individuals in the Veterans Health Administration of the US Department of Veterans Affairs. Am J Epidemiol. 2021;190(9):1928-1934. doi:10.1093/aje/kwab102 34467408

[32] Ehrenfeld JM, Gottlieb KG, Beach LB, Monahan SE, Fabbri D. Development of a natural language processing algorithm to identify and evaluate transgender patients in electronic health record systems. Ethn Dis. 2019;29(suppl 2):441-450. doi:10.18865/ed.29.S2.441 31308617 PMC6604788

[33] Gerth J, Becerra-Culqui T, Bradlyn A, . Agreement between medical records and self-reports: implications for transgender health research. Rev Endocr Metab Disord. 2018;19(3):263-269. doi:10.1007/s11154-018-9461-4 30219985 PMC6438197

[34] Liao KP, Cai T, Savova GK, . Development of phenotype algorithms using electronic medical records and incorporating natural language processing. BMJ. 2015;350:h1885. doi:10.1136/bmj.h1885 25911572 PMC4707569

[35] Xie F, Getahun D, Quinn VP, . An automated algorithm using free-text clinical notes to improve identification of transgender people. Inform Health Soc Care. 2021;46(1):18-28. doi:10.1080/17538157.2020.1828890 33203265

[36] Sedlakova J, Daniore P, Horn Wintsch A, ; University of Zurich Digital Society Initiative (UZH-DSI) Health Community. Challenges and best practices for digital unstructured data enrichment in health research: a systematic narrative review. PLOS Digit Health. 2023;2(10):e0000347. doi:10.1371/journal.pdig.0000347 37819910 PMC10566734

[37] Scheim AI, Coleman T, Lachowsky N, Bauer GR. Health care access among transgender and nonbinary people in Canada, 2019: a cross-sectional survey. CMAJ Open. 2021;9(4):E1213-E1222. doi:10.9778/cmajo.20210061 PMC8695530 34933879

[38] Scheim AI, Bauer GR. Sex and gender diversity among transgender persons in Ontario, Canada: results from a respondent-driven sampling survey. J Sex Res. 2015;52(1):1-14. doi:10.1080/00224499.2014.893553 24750105 PMC4299544

[39] Roberts CM, Klein DA, Adirim TA, Schvey NA, Hisle-Gorman E. Continuation of gender-affirming hormones among transgender adolescents and adults. J Clin Endocrinol Metab. 2022;107(9):e3937-e3943. doi:10.1210/clinem/dgac251 35452119

[40] Tonelli M, Wiebe N, Fortin M, ; Alberta Kidney Disease Network. Methods for identifying 30 chronic conditions: application to administrative data. BMC Med Inform Decis Mak. 2015;15(1):31. doi:10.1186/s12911-015-0155-5 25886580 PMC4415341

[41] Arcelus J, Bouman WP, Van Den Noortgate W, Claes L, Witcomb G, Fernandez-Aranda F. Systematic review and meta-analysis of prevalence studies in transsexualism. Eur Psychiatry. 2015;30(6):807-815. doi:10.1016/j.eurpsy.2015.04.005 26021270

[42] Statistics Canada. A generational portrait of Canada’s aging population from the 2021 census. Published April 27, 2022. Accessed November 7, 2024. https://www12.statcan.gc.ca/census-recensement/2021/as-sa/98-200-x/2021003/98-200-x2021003-eng.cfm

[43] Nolan IT, Kuhner CJ, Dy GW. Demographic and temporal trends in transgender identities and gender confirming surgery. Transl Androl Urol. 2019;8(3):184-190. doi:10.21037/tau.2019.04.09 31380225 PMC6626314

[44] Brown A. About 5% of young adults in the U.S. say their gender is different from their sex assigned at birth. Pew Research Center. Published June 27, 2022. Accessed November 7, 2024. https://www.pewresearch.org/short-reads/2022/06/07/about-5-of-young-adults-in-the-u-s-say-their-gender-is-different-from-their-sex-assigned-at-birth/

[45] Herman J, Flores A, O’Neill K. How many adults and youth identify as transgender in the United States? UCLA Williams Institute. Published June 2022. Accessed November 7, 2024. https://williamsinstitute.law.ucla.edu/wp-content/uploads/Trans-Pop-Update-Jun-2022.pdf

[46] Holzberg J, Ellis R, Virgile M, Assessing the feasibility of asking about gender identity in the current population survey: results from focus groups with members of the transgender population. US Census Bureau. Published April 2, 2018. Accessed November 7, 2024. https://www.census.gov/content/dam/Census/library/working-papers/2018/adrm/rsm2018-05.pdf

[47] Canadian Institute for Health Information. Gender stratifier: guidance on measuring and reporting health inequalities. Published June 2022. Accessed November 7, 2024. https://www.cihi.ca/sites/default/files/document/measuring-health-inequalities-toolkit-gender-stratifier-en.pdf

