# Supplementary material for: Validating a Case Definition for Transgender Adults Using Administrative Data
Source: JAMA Netw Open. 2025 Jan 3;8(1):e2451700. doi: 10.1001/jamanetworkopen.2024.51700 (PMC11699535; doi:10.1001/jamanetworkopen.2024.51700)
Supplement: Supplement 1. — eTable 1. Data Sources and Identifiers for Case Definitions to Identify Transgender Women and Men in Alberta eTable 2. Case Definition Performance in Identification of Transgender Women and Men in Self-Identified Transgender Cohort eFigure 1. Incidence per 100,000 Person-Years for Transgender Women, by Case Definition eFigure 2. Incidence per 100,000 Person-Years for Transgender Men, by Case Definition eTable 3. Incidence of Transgender Women and Men per 100,000 Person-Years Over a 10-Year Period, by Case Definition [file jamanetwopen-e2451700-s001.pdf]

Supplemental Online Content

Rytz CL, King JA, Saad N, et al. Validating a case definition for transgender adults using administrative data. *JAMA Netw. Open*. 2024;7(12):e2451700. doi:10.1001/jamanetworkopen.2024.51700

eTable 1. Data Sources and Identifiers for Case Definitions to Identify Transgender Women and Men in Alberta
eTable 2. Case Definition Performance in Identification of Transgender Women and Men in Self-Identified Transgender Cohort
eFigure 1. Incidence per 100,000 Person-Years for Transgender Women, by Case Definition
eFigure 2. Incidence per 100,000 Person-Years for Transgender Men, by Case Definition
eTable 3. Incidence of Transgender Women and Men per 100,000 Person-Years Over a 10-Year Period, by Case Definition

This supplemental material has been provided by the authors to give readers additional information about their work.

© 2024 Rytz CL et al. *JAMA Network Open.*

eTable 1. Data Sources and Identifiers for Case Definitions to Identify Transgender Women and Men in Alberta

**Case Definition**	**Description**	**Data Sources**	**Identifiers**
Transgender Women-Specific Case Definitions			
Case Definition 1	Individuals assigned male sex in provincial registry & ≥2 dispensations of exogenous estrogen therapy	Provincial Registry<br>Pharmaceutical Information Network	DIN codes: 02225190; 02148587; 02148595; 02449048; 02449056; 02449064; 02414678; 02414686; 02414694; 02505215; 02505223; 02247499; 02231509; 02247500; 02424924; 02424835; 02424843; 02241835; 02241837; 02244002; 02245676; 02243999; 02244000; 02244001; 02238704; 02243722; 02243724; 02246969; 02246967; 02246968; 02043440; 00741930; 02043386.<br><br>ATC codes: G03AA15; G03AB07; G03AA09; G03AB05; G03AB08; G03AA16; G03AA12; G03CA03; G03CA53; G03CA01; G03AA01; G03AA10; G03AB06; G03AA07; G03AB03; G03AA03; G03AB02; G03AA17; G03AA08; G03AA04; G03AB01; G03AA14; G03AA13; G03AA05; G03AB04; G03AA11; G03AB09; G03AA06; G03AA02.
Case Definition 2	Individuals assigned male sex in provincial registry & ≥1 gender-related endocrine disorder ICD-9/ICD-10 code	Provincial Registry<br>Physicians Claims<br>Discharge Abstract Database<br>National Ambulatory Care Classification System	ICD-9 codes: 302.3; 302.5; 302.6; 302.8<br><br>ICD-10 codes: F64; F64.0; F64.1; F64.2; F64.8; F64.9; F65.1; Z87.890; Z87.8; Z41.8; Z81.8; Z86.5; F68.8; Z92.28.
Case Definition 3	Individuals assigned male sex in provincial registry & ≥2 dispensations of cyproterone acetate	Provincial Registry<br>Pharmaceutical Information Network	DIN codes: 02245898; 02390760; 02395797; 02425017; 02309556.
Case Definition 4	Individuals assigned male sex in provincial registry & ≥2 dispensations of GnRH agonist	Provincial Registry<br>Pharmaceutical Information Network	DIN codes: 02240000; 02243856; 02412322; 02049325; 02225905; 00884502; 00836273; 02239834

© 2024 Rytz CL et al. *JAMA Network Open.*

Case Definition 5	Individuals assigned male sex in provincial registry & ≥2 dispensations of progestin	Provincial Registry<br>Pharmaceutical Information Network	DIN codes: 02166704; 02523493; 00729973; 00708917; 00030937; 00030848; 00585092; 0244682; 002267640; 02277298; 02244726; 02244727; 02493578; 02241013; 02247581; 02247582; 02247583; 02334992; 02403404; 02229840; 02229838; 02229839; 02128470; 02515504; 02253550; 02253577; 02322250; 02212056; 02212064; 02212072; 02252740; 02252759; 02231768; 02231769; 02231770; 02246627; 02246628; 02246629; 02476576; 02480247; 00739952; 02242878; 02242879; 02242880; 02239825; 02239826; 02239827; 02516187; 01977652; 02531828; 02166704; 00030945; 00729973; 00708917; 00030937; 02010933; 02010739; 02148552; 02148560; 02148579; 02463113; 02519062; 02221284; 02221292; 02221306; 02439913; 02462818.
Case Definition 6	Individuals assigned male sex in provincial registry with gender marker changed to female sex in provincial registry	Provincial Registry	-
Case Definition 7	Individuals assigned male sex in provincial registry with gender marker changed to "U" in provincial registry	Provincial Registry	-
Case Definition 8	Individuals assigned male sex in provincial registry with gender marker changed to "X" in provincial registry	Provincial Registry	-
Case Definition 9	Individuals assigned male sex in provincial registry with ≥1 procedural code specific for gender-affirmation	Provincial Registry<br>Discharge Abstract Database	CCI codes: 1.QD.^^.^^; 1.QE.^^.^^; 1.QG.^^.^^; 1.QH.^^.^^; 1.QJ.^^.^^; 1.QM.^^.^^; 1.QN.^^.^^; 1.QP.^^.^^; 1.QQ.^^.^^; 1.QT.^^.^^.<br><br>ICD-10 codes: Z90.7; N45.92; N45.02; N45.91; N45.01.
**Case Definition**	**Description**	**Data Sources**	**Identifiers**
Transgender Men-Specific Case Definitions			

© 2024 Rytz CL et al. *JAMA Network Open.*

Case Definition 1	Individuals assigned female sex in provincial registry & ≥2 dispensations of exogenous testosterone therapy	Provincial Registry<br>Pharmaceutical Information Network	DIN codes: 02245345; 02245346; 02249499; 00029246; 00030783; 02450550; 02322498; 02421186; 02496003; 02463792; 02463806; 02280248; 01977601; 01977571; 00782327; 02239653; 02245972; 02382369; 00739944; 02246063.<br><br>ATC codes: G0BA02; G03EK01; G03BA03.
Case Definition 2	Individuals assigned female sex in provincial registry & ≥1 gender-related endocrine disorder ICD-9/ICD-10 code	Provincial Registry<br>Physicians Claims<br>Discharge Abstract Database<br>National Ambulatory Care Classification System	ICD-9 codes: 302.3; 302.5; 302.6; 302.8<br><br>ICD-10 codes: F64; F64.0; F64.1; F64.2; F64.8; F64.9; F65.1; Z87.890; Z87.8; Z41.8; Z81.8; Z86.5; F68.8; Z92.28.
Case Definition 3	Individuals assigned female sex in provincial registry with gender marker changed to male sex in provincial registry	Provincial Registry	-
Case Definition 4	Individuals assigned female sex in provincial registry with gender marker changed to "U" in provincial registry	Provincial Registry	-
Case Definition 5	Individuals assigned female sex in provincial registry with gender marker changed to "X" in provincial registry	Provincial Registry	-
Case Definition 6	Individuals assigned female sex in provincial registry with ≥1 procedural code specific for gender-affirmation	Provincial Registry<br>Discharge Abstract Database	CCI codes: 1.RB.^^.^^; 1.RD.^^.^^; 1.RF.^^.^^; 1.RM.^^.^^; 1.RN.^^.^^; 1.RS.^^.^^; 1.RW.^^.^^ ; 1.RZ.^^.^^; 1.QE.84.^^; 1.SY.84.^^; 1.YM.89.^^; 1.YM.91.^^.<br><br>ICD-10 codes: Z41.1; Z96.8; Z90.7; Z12.8; N99.3; Y83.6.

© 2024 Rytz CL et al. *JAMA Network Open.*

eTable 2. Case Definition Performance in Identification of Transgender Women and Men in Self-Identified Transgender Cohort

Case Definition	Description	Sensitivity, % (95% CI)	PPV, % (95% CI)
Transgender Women			
Case Definition 1	male & ≥2 estrogen prescriptions	71.8 (63.7, 79.1)	5.0 (4.1, 6.0)
Case Definition 2	male & ≥1 gender-related ICD coding	85.2 (78.3, 90.6)	0.6 (0.5, 0.7)
Case Definition 3	male & ≥2 CPA prescriptions	7.7 (3.9, 13.4)	1.9 (1.0, 3.4)
Case Definition 4	male & ≥2 GnRHa prescriptions	0.7 (0.0, 3.9)	0.6 (0.0, 3.4)
Case Definition 5	male & ≥2 progestin prescriptions	17.6 (11.7, 24.9)	2.7 (1.7, 3.9)
Case Definition 6	male to female gender marker change	26.8 (19.7, 34.8)	2.3 (1.7, 3.2)
Case Definition 7	male to "U" gender marker change	-*	-*
Case Definition 8	male to "X" gender marker change	-*	-*
Case Definition 9	male with ≥1 gender-affirming ICD/CCI codes	4.9 (2.0, 9.9)	0.0 (0.0, 0.0)
Transgender Men			
Case Definition 1	female & ≥2 testosterone prescriptions	62.6 (55.0, 69.8)	2.4 (1.9, 2.8)
Case Definition 2	female & ≥1 gender-related ICD coding	77.0 (70.0, 83.0)	0.6 (0.5, 0.8)
Case Definition 3	female to male gender marker change	19.5 (13.9, 26.2)	2.7 (1.8, 3.7)
Case Definition 4	female to "U" gender marker change	-*	-*
Case Definition 5	female to "X" gender marker change	-*	-*
Case Definition 6	female with ≥1 gender-affirming ICD/CCI codes	0.6 (0.0, 3.2)	0.0 (0.0, 0.2)

*Notes: CI Confidence Interval; PPV Positive Predictive Value; ICD International Classification of Disease; CPA Cyproterone Acetate; GnRHa Gonadotropin-releasing Hormone Agonist; CCI Canadian Classification of Health Interventions. *Case definitions that identified <5 participants as transgender women or men were excluded to preserve anonymity.*

© 2024 Rytz CL et al. *JAMA Network Open.*

eFigure 1. Incidence per 100,000 Person-Years for Transgender Women, by Case Definition

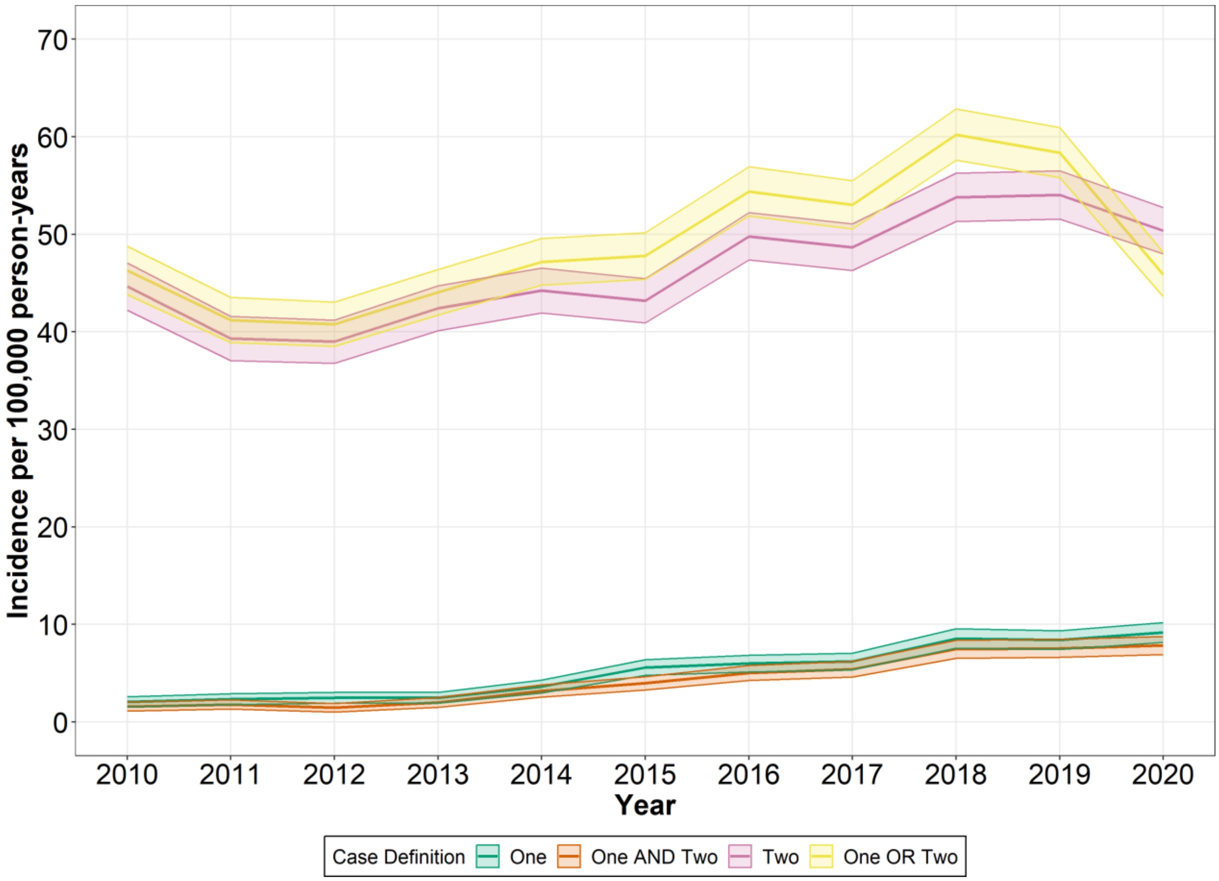

Midline represents incidence and boundaries represent 95% CI.

© 2024 Rytz CL et al. *JAMA Network Open.*

eFigure 2. Incidence per 100,000 Person-Years for Transgender Men, by Case Definition

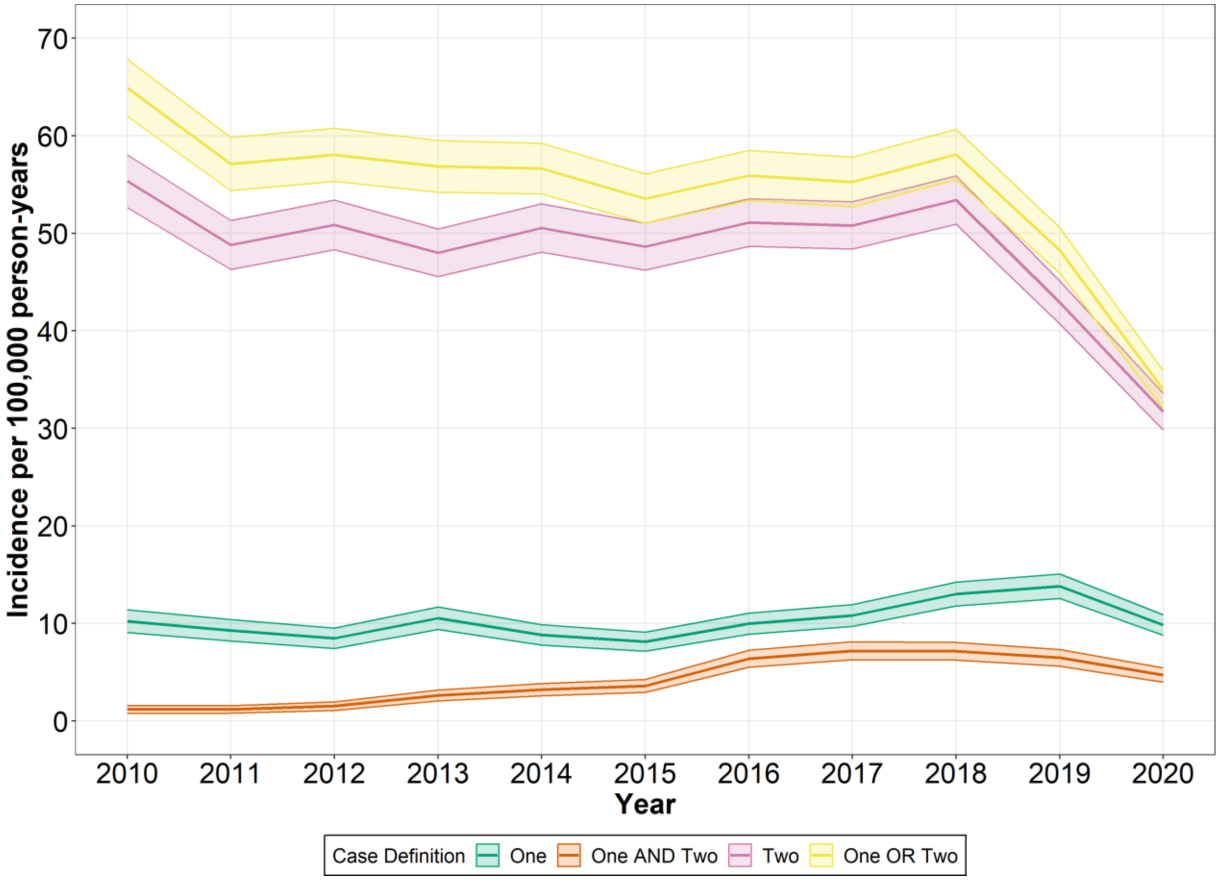

Midline represents incidence and boundaries represent 95% CI.

© 2024 Rytz CL et al. *JAMA Network Open.*

eTable 3. Incidence of Transgender Women and Men per 100,000 Person-Years Over a 10-Year Period, by Case Definition

		Incidence per 100,000 person-years, % (95% CI)	
**Year**	**Case Definition**	**Transgender Women**	**Transgender Men**
2010	1	2.06 (1.5, 2.6)	10.2 (9.1, 11.4)
	2	44.6 (42.2, 47.1)	55.4 (52.6, 58.1)
	1 AND 2	1.6 (1.1, 2.0)	1.2 (0.8, 1.6)
	1 OR 2	46.3 (43.8, 48.8)	64.9 (62.0, 67.8)
2011	1	2.3 (1.8, 2.9)	9.3 (8.2, 10.4)
	2	39.3 (37.0, 41.6)	48.8 (46.3, 51.3)
	1 AND 2	1.78 (1.3, 2.3)	1.1 (0.8, 1.6)
	1 OR 2	41.2 (38.9, 43.5)	57.1 (54.4, 59.8)
2012	1	2.5 (1.9, 3.0)	8.5 (7.4, 9.5)
	2	39.0 (36.7, 41.2)	50.9 (48.3, 53.4)
	1 AND 2	1.5 (1.0, 1.9)	1.5 (1.1, 2.0)
	1 OR 2	40.8 (38.5, 43.0)	58.0 (55.3, 60.8)
2013	1	2.5 (1.9, 3.0)	10.5 (9.4, 11.7)
	2	42.4 (40.1, 44.7)	48.0 (45.6, 50.4)
	1 AND 2	2.0 (1.5, 2.5)	2.6 (2.0, 3.2)
	1 OR 2	44.1 (41.7, 46.4)	56.9 (54.2, 59.5)
2014	1	3.6 (3.0, 4.3)	8.8 (7.8, 9.8)
	2	44.2 (41.9, 46.5)	50.5 (48.1, 53.0)
	1 AND 2	3.2 (2.6, 3.8)	3.2 (2.6, 3.8)
	1 OR 2	47.2 (44.8, 49.6)	56.6 (54.0, 59.2)
2015	1	5.6 (4.8, 6.4)	8.1 (7.1, 9.1)
	2	43.2 (40.9, 45.5)	48.6 (46.2, 51.0)
	1 AND 2	4.0 (3.3, 4.7)	3.6 (2.9, 4.3)
	1 OR 2	47.8 (45.4, 50.2)	53.6 (51.0, 56.1)
2016	1	6.0 (5.1, 6.8)	10.0 (8.9, 11.1)
	2	49.8 (47.4, 52.2)	51.1 (48.7, 53.6)
	1 AND 2	5.0 (4.3, 5.8)	6.4 (5.5, 7.3)
	1 OR 2	54.4 (51.9, 56.9)	55.9 (53.4, 58.5)
2017	1	6.2 (5.3, 7.0)	10.8 (9.7, 11.9)
	2	48.7 (46.3, 51.0)	50.8 (48.4, 53.2)
	1 AND 2	5.4 (4.6, 6.2)	7.2 (6.3, 8.1)
	1 OR 2	53.0 (50.5, 55.5)	55.3 (52.7, 57.8)
2018	1	8.6 (7.6, 9.6)	13.0 (11.8, 14.2)

© 2024 Rytz CL et al. *JAMA Network Open.*

	2	53.8 (51.3, 56.3)	53.4 (50.9, 55.9)
	1 AND 2	7.4 (6.5, 8.4)	7.2 (6.2, 8.1)
	1 OR 2	60.2 (57.6, 62.9)	58.1 (55.5, 60.6)
2019	1	8.4 (7.4, 9.4)	13.8 (12.6, 15.1)
	2	54.0 (51.6, 56.5)	42.9 (40.7, 45.1)
	1 AND 2	7.5 (6.6, 8.5)	6.5 (5.6, 7.3)
	1 OR 2	58.4 (55.8, 60.9)	48.2 (45.9, 50.6)
2020	1	9.2 (8.2, 10.2)	9.8 (8.8, 10.9)
	2	50.3 (48.0, 52.7)	31.7 (29.8, 33.6)
	1 AND 2	7.8 (6.9, 8.8)	4.7 (4.0, 5.4)
	1 OR 2	45.9 (43.6, 48.1)	34.0 (32.0, 35.9)

© 2024 Rytz CL et al. *JAMA Network Open.*
