# Supplementary material for: Validating a Case Definition for Transgender Adults Using Administrative Data
Source: JAMA Netw Open. 2025 Jan 3;8(1):e2451700. doi: 10.1001/jamanetworkopen.2024.51700 (PMC11699535; doi:10.1001/jamanetworkopen.2024.51700)
Supplement: Supplement 2. — Data Sharing Statement [file jamanetwopen-e2451700-s002.pdf]

Data Sharing Statement

Rytz. Validating a Case Definition for Transgender Adults Using Administrative Data. *JAMA Netw Open*. Published December 19, 2024. doi:10.1001/jamanetworkopen.2024.51700

Data
Data available: No

Additional Information
Explanation for why data not available: Data may be obtained from a third party and are not publicly available. Restrictions apply to the availability of these data. Data were obtained from Alberta Health Services and Alberta Health and may be available with the permission of Alberta Health Services and Alberta Health, respectively.
